# 4-Chloro-*N*′-(2-hy­droxy-4-meth­oxy­benzyl­idene)benzohydrazide methanol monosolvate

**DOI:** 10.1107/S1600536811039778

**Published:** 2011-10-05

**Authors:** Feng Zhi, Rong Wang, Yi Zhang, Qiang Wang, Yi-Lin Yang

**Affiliations:** a Third Affiliated Hospital of Suzhou University, Changzhou 213000, People’s Republic of China

## Abstract

The title compound, C_15_H_13_ClN_2_O_3_·CH_3_OH, was synthesized by the condensation reaction of 2-hy­droxy-4-meth­oxy­benzaldehyde with 4-chloro­benzohydrazide in methanol. The Schiff base mol­ecule displays a *trans* configuration with respect to the C=N and C—N bonds. The dihedral angle between the two benzene rings is 5.3 (2)°. In the crystal, mol­ecules are linked by N—H⋯O and O—H⋯O hydrogen-bond inter­actions into chains running parallel to the *a* axis. An intra­molecular O—H⋯N hydrogen bond is observed.

## Related literature

For background to Schiff base compounds, see: Fan *et al.* (2007 ▶); Kim *et al.* (2005 ▶); Nimitsiriwat *et al.* (2004 ▶). For their biological activity, see: Chen *et al.* (1997 ▶); Ren *et al.* (2002 ▶). For related structures, see: Mohd Lair *et al.* (2009 ▶); Fun *et al.* (2008 ▶); Yang (2008 ▶); Zhi (2008 ▶, 2009 ▶); Zhi & Yang (2007 ▶).
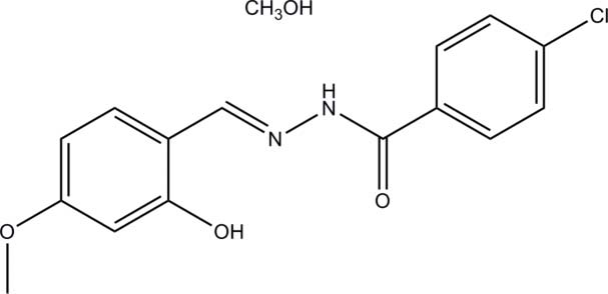


## Experimental

### 

#### Crystal data

C_15_H_13_ClN_2_O_3_·CH_4_O
*M* *_r_* = 336.77
Triclinic, 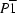

*a* = 6.570 (2) Å
*b* = 10.343 (3) Å
*c* = 12.707 (3) Å
α = 100.371 (2)°
β = 91.864 (2)°
γ = 101.663 (2)°
*V* = 829.7 (4) Å^3^
*Z* = 2
Mo *K*α radiation
μ = 0.25 mm^−1^
*T* = 298 K
0.17 × 0.13 × 0.12 mm

#### Data collection

Bruker SMART 1000 CCD area-detector diffractometer
Absorption correction: multi-scan (*SADABS*; Sheldrick, 1996 ▶) *T*_min_ = 0.959, *T*_max_ = 0.971
5945 measured reflections
3022 independent reflections
1724 reflections with *I* > 2σ(*I*)
*R*_int_ = 0.038

#### Refinement

*R*[*F* ^2^ > 2σ(*F* ^2^)] = 0.051
*wR*(*F* ^2^) = 0.144
*S* = 1.01
3022 reflections
216 parameters
1 restraint
H atoms treated by a mixture of independent and constrained refinement
Δρ_max_ = 0.23 e Å^−3^
Δρ_min_ = −0.17 e Å^−3^

### 

Data collection: *SMART* (Bruker, 2002 ▶); cell refinement: *SAINT* (Bruker, 2002 ▶); data reduction: *SAINT*; program(s) used to solve structure: *SHELXS97* (Sheldrick, 2008 ▶); program(s) used to refine structure: *SHELXL97* (Sheldrick, 2008 ▶); molecular graphics: *SHELXTL* (Sheldrick, 2008 ▶); software used to prepare material for publication: *SHELXTL*.

## Supplementary Material

Crystal structure: contains datablock(s) global, I. DOI: 10.1107/S1600536811039778/rz2644sup1.cif

Structure factors: contains datablock(s) I. DOI: 10.1107/S1600536811039778/rz2644Isup2.hkl

Supplementary material file. DOI: 10.1107/S1600536811039778/rz2644Isup3.cml

Additional supplementary materials:  crystallographic information; 3D view; checkCIF report

## Figures and Tables

**Table 1:** Hydrogen-bond geometry (Å, °)

*D*—H⋯*A*	*D*—H	H⋯*A*	*D*⋯*A*	*D*—H⋯*A*
O4—H4⋯O2^i^	0.82	1.83	2.646 (3)	177
N2—H2⋯O4	0.90 (1)	2.00 (1)	2.876 (3)	163 (2)
O1—H1⋯N1	0.82	1.96	2.676 (3)	146

Symmetry code: (i) 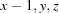.

**Fig. 1.**
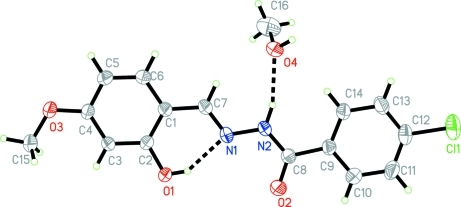
The molecular structure of the title compound with displacement ellipsoids drawn at the 30% probability level. Intramolecular O—H···N hydrogen bond is shown as a dashed line.

**Fig. 2.**
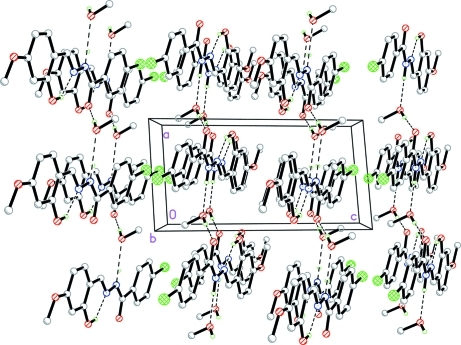
Crystal packing of the title compound, viewed along the a axis. Intermolecular hydrogen bonds are shown as dashed lines.

## supplementary crystallographic information

### Comment

In recent years, much attention has been focused on the synthesis, structures, and properties of Schiff base compounds (Fan *et al.*, 2007; Kim *et al.*, 2005; Nimitsiriwat *et al.*, 2004). Some of the compounds have been found to have excellent pharmacological and antibacterial activity (Chen *et al.*, 1997; Ren *et al.*, 2002). We report herein the crystal structure of the title new Schiff base compound (Fig. 1) derived from the condensation reaction of 2-hydroxy-4-methoxybenzaldehyde with 4-chlorobenzohydrazide.

The asymmetric unit of the title compound contains a Schiff base molecule and a methanol molecule of crystallization. The Schiff base molecule displays a *trans* configuration with respect to the C═N and C–N bonds. There is an intramolecular O—H···N hydrogen bond in the molecule. The dihedral angle between the two benzene rings is 5.3 (2)°. All the bond lengths are within normal ranges and comparable to those in other similar compounds (Mohd Lair *et al.*, 2009; Fun *et al.*, 2008; Yang, 2008; Zhi, 2008; Zhi & Yang, 2007; Zhi, 2009). In the crystal (Fig. 2), molecules are linked by N—H···O and O—H···O hydrogen interactions into one-dimensional chains along the *a* axis (Table 1).

### Experimental

2-Hydroxy-4-methoxybenzaldehyde (0.01 mol, 1.52 g) and 4-chlorobenzohydrazide (0.01 mol, 1.71 g) were dissolved in methanol (50 ml). The mixture was stirred at room temperature to give a clear colourless solution. Crystals of the title compound were formed by slow evaporation of the solvent for several days at room temperature.

### Refinement

Atom H2 was located in a difference Fourier map and refined with the N—H distance restrained to 0.90 (1) Å. All other H atoms were positioned geometrically [C—H = 0.93–0.96 Å, O—H = 0.82 Å] and refined using a riding model, with *U*_iso_(H) = 1.2*U*_eq_(C) or 1.5*U*_eq_(O, C) for hydroxy and methyl H atoms.

### Crystal data

**Table:**

C_15_H_13_ClN_2_O_3_·CH_4_O	*Z* = 2
*M**_r_* = 336.77	*F*(000) = 352
Triclinic, *P*1	*D*_x_ = 1.348 Mg m^−^^3^
Hall symbol: -P 1	Mo *K*α radiation, λ = 0.71073 Å
*a* = 6.570 (2) Å	Cell parameters from 1241 reflections
*b* = 10.343 (3) Å	θ = 2.3–24.5°
*c* = 12.707 (3) Å	µ = 0.25 mm^−^^1^
α = 100.371 (2)°	*T* = 298 K
β = 91.864 (2)°	Block, colorless
γ = 101.663 (2)°	0.17 × 0.13 × 0.12 mm
*V* = 829.7 (4) Å^3^	

### Data collection

**Table:**

Bruker SMART 1000 CCD area-detector diffractometer	3022 independent reflections
Radiation source: fine-focus sealed tube	1724 reflections with *I* > 2σ(*I*)
graphite	*R*_int_ = 0.038
ω scans	θ_max_ = 25.5°, θ_min_ = 2.4°
Absorption correction: multi-scan (*SADABS*; Sheldrick, 1996)	*h* = −7→7
*T*_min_ = 0.959, *T*_max_ = 0.971	*k* = −12→12
5945 measured reflections	*l* = −15→13

### Refinement

**Table:**

Refinement on *F*^2^	Primary atom site location: structure-invariant direct methods
Least-squares matrix: full	Secondary atom site location: difference Fourier map
*R*[*F*^2^ > 2σ(*F*^2^)] = 0.051	Hydrogen site location: inferred from neighbouring sites
*wR*(*F*^2^) = 0.144	H atoms treated by a mixture of independent and constrained refinement
*S* = 1.01	*w* = 1/[σ^2^(*F*_o_^2^) + (0.0633*P*)^2^] where *P* = (*F*_o_^2^ + 2*F*_c_^2^)/3
3022 reflections	(Δ/σ)_max_ < 0.001
216 parameters	Δρ_max_ = 0.23 e Å^−^^3^
1 restraint	Δρ_min_ = −0.17 e Å^−^^3^

### Special details

**Table:**

Geometry. All e.s.d.'s (except the e.s.d. in the dihedral angle between two l.s. planes) are estimated using the full covariance matrix. The cell e.s.d.'s are taken into account individually in the estimation of e.s.d.'s in distances, angles and torsion angles; correlations between e.s.d.'s in cell parameters are only used when they are defined by crystal symmetry. An approximate (isotropic) treatment of cell e.s.d.'s is used for estimating e.s.d.'s involving l.s. planes.
Refinement. Refinement of *F*^2^ against ALL reflections. The weighted *R*-factor *wR* and goodness of fit *S* are based on *F*^2^, conventional *R*-factors *R* are based on *F*, with *F* set to zero for negative *F*^2^. The threshold expression of *F*^2^ > σ(*F*^2^) is used only for calculating *R*-factors(gt) *etc*. and is not relevant to the choice of reflections for refinement. *R*-factors based on *F*^2^ are statistically about twice as large as those based on *F*, and *R*- factors based on ALL data will be even larger.

### Fractional atomic coordinates and isotropic or equivalent isotropic displacement parameters (Å^2^)

**Table:**

	*x*	*y*	*z*	*U*_iso_*/*U*_eq_	
Cl1	0.45941 (17)	1.34163 (8)	0.04723 (7)	0.1057 (4)	
N1	0.6218 (3)	0.69152 (19)	0.29276 (16)	0.0550 (6)	
N2	0.5628 (3)	0.7970 (2)	0.25424 (18)	0.0544 (6)	
O1	0.8829 (3)	0.55918 (18)	0.37185 (18)	0.0764 (6)	
H1	0.8513	0.6186	0.3438	0.115*	
O2	0.8950 (3)	0.91058 (17)	0.25849 (18)	0.0853 (7)	
O3	0.5767 (3)	0.14043 (16)	0.46539 (16)	0.0715 (6)	
O4	0.1182 (3)	0.72464 (19)	0.20964 (17)	0.0731 (6)	
H4	0.0462	0.7810	0.2227	0.110*	
C1	0.5106 (4)	0.4800 (2)	0.34710 (19)	0.0485 (6)	
C2	0.7089 (4)	0.4662 (2)	0.3793 (2)	0.0506 (6)	
C3	0.7361 (4)	0.3544 (2)	0.42035 (19)	0.0528 (7)	
H3	0.8687	0.3472	0.4431	0.063*	
C4	0.5660 (4)	0.2544 (2)	0.4272 (2)	0.0533 (7)	
C5	0.3685 (4)	0.2649 (3)	0.3942 (2)	0.0652 (8)	
H5	0.2542	0.1964	0.3976	0.078*	
C6	0.3423 (4)	0.3768 (3)	0.3565 (2)	0.0634 (8)	
H6	0.2084	0.3845	0.3366	0.076*	
C7	0.4739 (4)	0.5964 (2)	0.30752 (19)	0.0538 (7)	
H7	0.3371	0.6024	0.2921	0.065*	
C8	0.7072 (4)	0.9011 (2)	0.2375 (2)	0.0565 (7)	
C9	0.6350 (4)	1.0083 (2)	0.1913 (2)	0.0526 (7)	
C10	0.7810 (5)	1.1221 (3)	0.1836 (2)	0.0669 (8)	
H10	0.9185	1.1298	0.2084	0.080*	
C11	0.7275 (5)	1.2247 (3)	0.1399 (2)	0.0759 (9)	
H11	0.8277	1.3008	0.1355	0.091*	
C12	0.5255 (5)	1.2130 (3)	0.1031 (2)	0.0680 (8)	
C13	0.3780 (5)	1.1019 (3)	0.1095 (2)	0.0761 (9)	
H13	0.2408	1.0951	0.0846	0.091*	
C14	0.4322 (4)	0.9993 (3)	0.1531 (2)	0.0699 (8)	
H14	0.3311	0.9233	0.1567	0.084*	
C15	0.7748 (4)	0.1250 (3)	0.5037 (3)	0.0789 (9)	
H15A	0.8623	0.1154	0.4449	0.118*	
H15B	0.7574	0.0465	0.5356	0.118*	
H15C	0.8382	0.2028	0.5563	0.118*	
C16	0.0297 (6)	0.6290 (4)	0.1191 (3)	0.1093 (12)	
H16A	0.0040	0.6737	0.0616	0.164*	
H16B	−0.0994	0.5770	0.1358	0.164*	
H16C	0.1236	0.5706	0.0978	0.164*	
H2	0.4259 (18)	0.792 (3)	0.240 (2)	0.085 (10)*	

### Atomic displacement parameters (Å^2^)

**Table:**

	*U*^11^	*U*^22^	*U*^33^	*U*^12^	*U*^13^	*U*^23^
Cl1	0.1478 (9)	0.0832 (6)	0.1065 (7)	0.0441 (6)	0.0043 (6)	0.0498 (5)
N1	0.0538 (13)	0.0482 (12)	0.0692 (14)	0.0191 (11)	−0.0014 (11)	0.0188 (10)
N2	0.0448 (13)	0.0522 (12)	0.0731 (15)	0.0169 (11)	−0.0001 (11)	0.0227 (11)
O1	0.0478 (11)	0.0667 (12)	0.1210 (17)	−0.0033 (9)	−0.0109 (11)	0.0546 (11)
O2	0.0489 (12)	0.0643 (12)	0.148 (2)	0.0151 (10)	−0.0058 (12)	0.0321 (12)
O3	0.0582 (12)	0.0592 (11)	0.1055 (15)	0.0078 (9)	0.0026 (11)	0.0437 (10)
O4	0.0475 (11)	0.0716 (12)	0.1049 (16)	0.0173 (9)	−0.0006 (11)	0.0252 (11)
C1	0.0453 (15)	0.0491 (13)	0.0541 (16)	0.0143 (12)	0.0040 (12)	0.0129 (12)
C2	0.0464 (16)	0.0455 (13)	0.0609 (16)	0.0059 (12)	0.0044 (12)	0.0169 (12)
C3	0.0424 (15)	0.0513 (14)	0.0698 (18)	0.0128 (12)	0.0006 (13)	0.0226 (12)
C4	0.0535 (17)	0.0463 (14)	0.0641 (17)	0.0096 (13)	0.0067 (13)	0.0217 (12)
C5	0.0448 (16)	0.0615 (16)	0.093 (2)	0.0056 (13)	0.0075 (15)	0.0307 (15)
C6	0.0418 (15)	0.0650 (17)	0.089 (2)	0.0121 (13)	0.0052 (14)	0.0290 (15)
C7	0.0476 (16)	0.0541 (15)	0.0650 (18)	0.0177 (13)	0.0027 (13)	0.0175 (13)
C8	0.0472 (17)	0.0488 (15)	0.0755 (19)	0.0152 (13)	0.0041 (14)	0.0113 (13)
C9	0.0525 (16)	0.0473 (14)	0.0616 (17)	0.0157 (12)	0.0072 (13)	0.0133 (12)
C10	0.0622 (18)	0.0604 (17)	0.077 (2)	0.0061 (15)	−0.0002 (15)	0.0198 (15)
C11	0.091 (2)	0.0552 (17)	0.081 (2)	0.0040 (16)	0.0001 (18)	0.0276 (15)
C12	0.096 (3)	0.0573 (17)	0.0602 (18)	0.0267 (17)	0.0105 (17)	0.0236 (14)
C13	0.0632 (19)	0.080 (2)	0.102 (2)	0.0287 (16)	0.0104 (17)	0.0439 (18)
C14	0.0574 (19)	0.0624 (17)	0.100 (2)	0.0135 (14)	0.0084 (16)	0.0408 (16)
C15	0.071 (2)	0.0640 (17)	0.110 (2)	0.0128 (16)	−0.0042 (18)	0.0434 (17)
C16	0.090 (3)	0.138 (3)	0.095 (3)	0.024 (2)	−0.001 (2)	0.012 (2)

### Geometric parameters (Å, °)

**Table:**

Cl1—C12	1.736 (3)	C5—H5	0.9300
N1—C7	1.280 (3)	C6—H6	0.9300
N1—N2	1.390 (3)	C7—H7	0.9300
N2—C8	1.337 (3)	C8—C9	1.492 (3)
N2—H2	0.901 (10)	C9—C10	1.380 (3)
O1—C2	1.355 (3)	C9—C14	1.383 (4)
O1—H1	0.8200	C10—C11	1.382 (4)
O2—C8	1.235 (3)	C10—H10	0.9300
O3—C4	1.366 (3)	C11—C12	1.367 (4)
O3—C15	1.423 (3)	C11—H11	0.9300
O4—C16	1.398 (3)	C12—C13	1.362 (4)
O4—H4	0.8200	C13—C14	1.383 (4)
C1—C2	1.395 (3)	C13—H13	0.9300
C1—C6	1.397 (3)	C14—H14	0.9300
C1—C7	1.445 (3)	C15—H15A	0.9600
C2—C3	1.392 (3)	C15—H15B	0.9600
C3—C4	1.378 (3)	C15—H15C	0.9600
C3—H3	0.9300	C16—H16A	0.9600
C4—C5	1.382 (4)	C16—H16B	0.9600
C5—C6	1.369 (3)	C16—H16C	0.9600
			
C7—N1—N2	116.3 (2)	C10—C9—C14	117.9 (2)
C8—N2—N1	120.2 (2)	C10—C9—C8	118.0 (2)
C8—N2—H2	121.7 (18)	C14—C9—C8	124.1 (2)
N1—N2—H2	118.1 (18)	C9—C10—C11	121.5 (3)
C2—O1—H1	109.5	C9—C10—H10	119.2
C4—O3—C15	118.4 (2)	C11—C10—H10	119.2
C16—O4—H4	109.5	C12—C11—C10	119.2 (3)
C2—C1—C6	117.5 (2)	C12—C11—H11	120.4
C2—C1—C7	122.8 (2)	C10—C11—H11	120.4
C6—C1—C7	119.7 (2)	C13—C12—C11	120.6 (3)
O1—C2—C3	116.9 (2)	C13—C12—Cl1	120.3 (3)
O1—C2—C1	122.3 (2)	C11—C12—Cl1	119.1 (2)
C3—C2—C1	120.8 (2)	C12—C13—C14	120.0 (3)
C4—C3—C2	119.7 (2)	C12—C13—H13	120.0
C4—C3—H3	120.1	C14—C13—H13	120.0
C2—C3—H3	120.1	C9—C14—C13	120.8 (3)
O3—C4—C3	124.1 (2)	C9—C14—H14	119.6
O3—C4—C5	115.4 (2)	C13—C14—H14	119.6
C3—C4—C5	120.4 (2)	O3—C15—H15A	109.5
C6—C5—C4	119.5 (2)	O3—C15—H15B	109.5
C6—C5—H5	120.3	H15A—C15—H15B	109.5
C4—C5—H5	120.3	O3—C15—H15C	109.5
C5—C6—C1	122.0 (2)	H15A—C15—H15C	109.5
C5—C6—H6	119.0	H15B—C15—H15C	109.5
C1—C6—H6	119.0	O4—C16—H16A	109.5
N1—C7—C1	122.7 (2)	O4—C16—H16B	109.5
N1—C7—H7	118.6	H16A—C16—H16B	109.5
C1—C7—H7	118.6	O4—C16—H16C	109.5
O2—C8—N2	122.4 (2)	H16A—C16—H16C	109.5
O2—C8—C9	119.8 (2)	H16B—C16—H16C	109.5
N2—C8—C9	117.8 (2)		

### Hydrogen-bond geometry (Å, °)

**Table:**

*D*—H···*A*	*D*—H	H···*A*	*D*···*A*	*D*—H···*A*
O4—H4···O2^i^	0.82	1.83	2.646 (3)	177.
N2—H2···O4	0.90 (1)	2.00 (1)	2.876 (3)	163 (2)
O1—H1···N1	0.82	1.96	2.676 (3)	146.

Symmetry codes: (i) *x*−1, *y*, *z*.
